# Collectanea Medica

**Published:** 1813-10

**Authors:** 


### COLLECTANEA MEDICA,

CONSISTING OF

### ANECDOTES, FACTS, EXTRACTS, ILLUSTRATIONS, QUERIES, SUGGESTIONS, &c.

RELATING TO THE

*History or the Art of Medicine, and the Auxiliary Sciences.*

---

### *On the Heat evolved during Inflammation of the Human Body.*
By THOMAS THOMSON, M.D. F.R.S.

THAT the heat evolved by the human body is very considerable, and that in cases of inflammation this heat is very much increased, are facts with which every body is acquainted; but I am ignorant of any attempt hitherto made to estimate the increase of heat that is given off in cases of inflammation. On that account I think it worth while to record an observation which I had an opportunity of making upon myself, during the course of last winter. It is far from determining the whole heat given off during the inflammation; but as it is at least an approach towards accuracy, and as I was at as much pains as possible, considering the situation in which I was at the time, I conceive the statement will add another and a curious fact to animal physiology.

During the month of January last, in consequence of walking about in rainy weather in thin shoes for a considerable part of the day, and afterwards sitting for several hours with wet feet, I caught a violent cold, which was attended with fever, and among other inflammatory symptoms a throbbing pain took place in the right groin, accompanied with swelling of the inguinal glands. To prevent this pain from proceeding to suppuration, I applied, for four days successively, and 36 times each day, two cotton cloths successively wrung out of cold water to the swelled part. The average temperature of the cold water employed was 40°. The cloths were removed when they felt hot; and from several trials this appeared to indicate a temperature of about 90°; so that each cloth, and the water which it contained, was heated at an average 50°.

The first cloth dry weighed----530 grains
The second ----------------- 458

The first when wet weighed 1459 or 929 water + 530 cloth
The second -------------- 1434 or 976 + 458

I made several experiments to determine the specific heat of cotton, but found it attended with unexpected difficulty.

 When

When cotton wool is employed it is so elastic and bulky that you are obliged to use a much smaller weight of it than of the hot water with which you mix it. This occasions great inaccuracy. When cotton cloth is employed, a considerable time elapses before you can mix it properly with the water, and this occasions uncertainty. I state, therefore, the results which I obtained with considerable hesitation. The specific heat of cotton, by my trials, is 0·53, that of water being 1. I shall therefore consider it as half as great as that of water.

We may, therefore, substitute for the two cotton cloths a quantity of water equal to half the weight of each. We may say, therefore, that 2399 grains of water were heated 50 degrees 18 times a day for four days together, making a total of 30 pounds troy heated 50° in the course of four days by the inflamed part. This is nearly the same quantity of heat that would have been requisite to heat 8½ lbs. of water from the temperature of 40° to that of 212°. This amounts nearly to seven wine pints.

So that in the course of four days this small inflamed spot gave out a quantity of heat sufficient to have heated seven wine pints of water from 40° to 212°; yet the temperature was not sensibly less than that of the rest of the body at the end of the experiment. The inflammation, however, was gone, and did not again return.

Nor was this quantity of heat, considerable as it was, the whole that was evolved. Some was lost by the evaporation of the moisture from the wet cloth, which must have taken place to a certain extent, and some must have made its escape during the night, when the wet cloths were applied very irregularly, and at long intervals.—*Annals of Philosophy.*

---

*Additional Observations on the Effects of Magnesia in preventing an increased Formation of Uric Acid; with Remarks on the Influence of Acids upon the Composition of the Urine.* By WILLIAM THOMAS BRANDE, Esq. F.R.S. Prof. Chem. R. I.—Read before the Royal Society, June 3, 1812.

In a paper which I had the honor of laying before this society, about three years ago, and which is published in the Philosophical Transactions,* some cases are related, illustrating the effects of magnesia in preventing an increased formation of uric acid, and some experiments are detailed, instituted with a view to discover its mode of action.

Since that period, many opportunities have occurred, both

* For 1813, p. 106.

to to Sir Everard Home and myself, of confirming its efficacy upon a more extended scale, and of ascertaining the efficient treatment of those cases in which magnesia is ineffectual, and in which it has even been found to aggravate the complaint.

To bring forward additional evidence in favor of the use of magnesia, and to distinguish the cases in which its use is indicated, from those where it is improper or hurtful, are the principal objects of the present communication, and will be considered in the two following sections.

### Section I.

The following is the case of a gentleman who suffered from a calculous complaint, during which he was accidentally induced to employ magnesia, the effects of which he has thus described.

Case I.—About twenty-seven years ago, I felt a pain in one of my kidnies, particularly when in bed, which continued to increase during six months. I had likewise an occasional sympathetic pain in the testicles, and violent and excruciating pains in the left kidney now became frequent. These attacks were sometimes brought on by stooping to take up something; but at other times without any apparent cause. They lasted from twelve to twenty-four hours, and I obtained some relief from the application of warm flannels; but they always left me languid and relaxed.

On the fourth attack I consulted a physician, who imagined that my complaint had been induced by drinking cyder, in which I had formerly indulged. He ordered me weak Hollands and water for common drink, and prescribed the lixivium of tartar to be taken in broth. This medicine was persevered in for some time; but I found it gradually weaken my stomach, and impair my digestive powers.

About nine months after my first attack in the kidney, I walked from Hampstead to London after dinner, and on the following day, I clearly felt something pass from the kidney to the bladder, and suspected what it was. I took about a pint of Hollands and water, and on attempting shortly afterwards to void my urine, found that the passage was blocked up, but had scarcely time to consider of my situation before the obstruction moved forwards to within an inch of the extremity of the urethra: it remained there till the following evening, when, by the help of a small pair of watchmaker's forceps, I succeeded in extracting a stone, which was the source of the mischief.

It was jagged and rough, and of a deep brick-red color. I afterwards voided a considerable quantity of red crystalline sand.

My

My physician, who was apprehensive of a return of the disorder, desired me to purchase of Cadell an anonymous pamphlet upon the Stone and Gravel, and to observe the rules there laid down. This treatise particularly recommended the use of the alkalies. I therefore took the lixivium, and two bottles of Perry's solvent; but the red deposit in my urine continued, my loins felt weak, and when in bed very painful.

Being in the profession of the law, and much employed, I was under the necessity of leading a very sedentary life, which so aggravated my tendency to bile and indigestion, that I seldom could get above two or three hours sleep.

With a view to alleviate these symptoms, and not with any idea of its being beneficial to the stone, I resorted to magnesia, which I continued with little intermission for eight months in the dose of a tea-spoonful or two, every evening before I went to bed. The long vacation coming on, I gradually took more exercise, and used the cold bath. The tone of my stomach, at the end of the period I have mentioned, was so far restored as to induce me to set medicine of all kinds aside, except when any food or drink disagrees, when I occasionally resort to the magnesia. Under such treatment, the weakness and pain in my kidney left me, and the red sand entirely disappeared. I have since enjoyed a very good state of health, and am now in my fifty-seventh year.

If I occasionally make a little free with the good things of this world, my stomach reminds me of the improper use of the lixivium, especially when I am prevented taking my usual exercise.

The above case is important, not only as furnishing a striking and unprejudiced instance of the effect of magnesia, in counteracting the tendency to form uric calculi and gravel; but likewise, as demonstrating its efficacy where the alkalies had failed, and where the digestive organs had been injured in consequence of the use of such remedies: the time which has elapsed since the cure of this and other cases, without a relapse, is also strongly in favor of this mode of treatment.

Case 2.—A gentleman, twenty years of age, who had suffered from heart-burn and other dyspeptic symptoms, was seized, on the 1st of June, 1811, with a violent pain in the loins, and more especially in the right kidney, and during the night he passed a large quantity of red sand with his urine. On the 2d, with a view to relieve the pain, which had increased considerably, he took fifty drops of laudanum, and drank freely of barley water. The night was passed

 more more quietly; but on the morning of the 3d he was seized with a violent pain in the kidney, and with the usual symptoms of the passage of a calculus along the ureter. These continued with more or less violence till the evening of the 4th, when he became perfectly easy, and remained so till the morning of the 6th, when, with considerable pain and difficulty, he voided a calculus composed of uric acid, weighing nine grains. For several successive days his urine deposited a large quantity of red sand, and three very small round calculi were voided.

He was now directed to abstain from all kinds of fermented liquors and sour food, and to take a pint of treble soda water (containing three drachms of sub-carbonate of soda) daily. Under this treatment he continued to recover, and remained perfectly free from complaint until the end of August, when a copious deposit of red sand appeared in his urine: he had little pain in the affected kidney, but complained of almost constant nausea, or want of appetite. The soda water was increased to a pint and a half, and afterwards to two pints daily, and in the intervals he drank very freely of barley water.

Having persevered in this way for ten days without receiving any benefit, he was induced to make a trial of magnesia, of which he took one tea-spoonful night and morning in cold chamomile tea. In about a week, the state of his stomach was much improved, and the deposit in the urine proportionally diminished; and in three weeks every symptom of disease had disappeared.

In February, 1812, having persevered in the use of magnesia with little intermission, I was informed that the sand had returned, that increasing the quantity of magnesia had produced no good effect, and that alkalies materially aggravated his complaint, by disagreeing with the stomach and greatly increasing the urinary deposit.

On examining the sand, I found that instead of consisting as formerly of uric acid, it was composed of a mixture of the ammoniaco-magnesian phosphate with phosphate of lime. He was directed to abstain from magnesia and alkalies, and to adopt a plan of treatment which it is the object of the second section of this paper more particularly to explain.

The foregoing is a well-marked case of uric gravel with a strong tendency to form calculi, materially relieved by the use of alkaline remedies: it illustrates their usual effects when carelessly persevered in, and shows the advantage with which magnesia may in such instances be employed: it also exhibits the effect of magnesia and the alkalies, in producing the deposit posit of *white sand* (or phosphates) in the urine, when the *red sand* (or uric acid) has been removed.

The cases which follow are selected, from among others, to explain the best mode of preventing the formation of white sand, and to show the most effectual treatment where it is a natural deposit in the urine, or where it has been induced by the incautious exhibition of alkaline medicines.

Section II.

The white sand so frequently voided by persons laboring under calculous complaints, was first analysed by Dr. Wollaston,* who found it composed of ammoniaco-magnesian phosphate, either alone or mixed with variable proportions of phosphate of lime. The use of acid medicines in these cases was also first suggested by the same able chemist; but although his valuable observations have been before the public for nearly fifteen years, I am not aware that any experiments have been made to ascertain what acids are best calculated to produce the desired effect, or to illustrate their mode of action.

Since my former communication, I have lost no opportunity of attending to this important subject, and hope that the conclusions, suggested by the following cases, will be deemed satisfactory, and that their application in practice may lead to useful results.

Case 1.—A gentleman, fifty years of age, who about ten years before had undergone the operation for the stone,† was attacked on the 14th of January, 1810, with violent pain in the right kidney and ureter, which lasted two days. On the 17th, these symptoms subsided, and were followed by those of stone in the bladder, which continued for some days; and although he had taken abundance of barley water and similar diluents, the stone showed no disposition to pass. On account of his former sufferings, this circumstance rendered him extremely uneasy; and on the evening of the 21st, he suffered several severe paroxysms of pain on attempting to make water. Under these circumstances, he was desired to take a purge, composed of two ounces of infusion of senna, two drachms of tincture of senna, and twenty grains of powdered jalap.‡ In three

---

* Phil. Trans. 1797.

† The stone extracted consisted of a nucleus of uric acid about the size of a pea, encrusted with a mixture of the phosphates. It was broken during the operation, but appeared to have been of the size of a pigeon's egg.

‡ I recommended this treatment in consequence of having heard Sir three hours this began to take powerful effect, and during the violence of the operation, he was so fortunate as to void the calculus with his urine: it weighed eight grains. On the 28th he again suffered pain in the region of the kidneys, and voided much sand, composed of uric acid, with ammoniaco-magnesian phosphate. He now took three half pints of soda-water daily, which materially increased the proportion of the triple phosphate, while that of uric acid was considerably diminished. Ten drops of muriatic acid were then taken three times a-day in water. The red sand now began to re-appear, and on the 4th of February he voided a very small uric calculus. The urine made after dinner contained more or less mucus streaked with blood, a symptom which was much aggravated by a slight excess in wine. On the 6th he left London, and employed no medicine until the 12th, when he returned in consequence of having voided a large quantity of the white sand.

Having observed the efficacy of carbonic acid in preventing the deposition of the phosphates, and having found it less liable than any other acid to induce a return of the uric gravel and calculi, I now directed him to take half a pint of water highly impregnated with fixed air, four or five times a-day, and to drink cyder instead of wine. On the 18th of February, his urine was less turbid than it had been for some months before, and on the 20th of March, having continued the use of carbonic acid, he had no remaining symptoms.*

In August his urine became again turbid, but by the use of vinegar and lemon juice at his meals, which acids, he now finds, have no tendency to induce a return of the red gravel, he succeeds in preventing this symptom.

Case 2.—On the 11th of October, 1812, the operation for stone in the bladder was performed upon a boy, eleven years of age, and four calculi were extracted, of which the largest was of the size of a small horse-bean: they were each composed of a nucleus or centre of uric acid, upon which the ammoniaco-magnesian phosphate was deposited.

---

Sir Everard Home state a case, in his Surgical Lectures, of a gentleman who suffered a bougie to pass so far into the urethra, that it could not be removed by any instrument. During the operation of a purge it was expelled with considerable force.

* I have several times examined the urine, with a view to ascertain whether any of the acids which were exhibited could be detected in that secretion; but the results of such experiments are so much interfered with by the very compound nature of the urine, that I have not hitherto been able to draw any satisfactory conclusions respecting them.

After the operation, the urine deposited a large quantity of white sediment, and some small pieces of red gravel were occasionally voided. He was now directed to take eight grains of citric acid dissolved in barley water, three times daily. Under this treatment the sediment in the urine was considerably diminished, but did not wholly disappear. The dose of the acid was gradually increased to twenty grains, by which means the sediment was only occasionally deposited, and consisted of little else than mucus. It was observed, that whenever the citric acid was omitted, even for twenty-four hours, the sediment was greatly increased, and this was constantly attended with frequent desire to make water, and other symptoms of irritation in the bladder. On resuming the use of the citric acid, the sediment always disappeared, and the irritation of the bladder subsided; and this happened so frequently, that no doubt could be entertained of the influence of the medicine on the composition of the urine.

This plan of treatment was continued for three months. At the end of that period, it was found that the urine had not the same disposition to deposit the phosphates as formerly: even when the medicine was omitted, the sediment was small in quantity, and not constant in its appearance. He was now directed to omit the use of the citric acid, and occasionally to eat oranges and other acid fruits. He continued this plan until the beginning of April, 1813: his urine was then quite clear, and he had no symptoms of disease.

Case 3.—In the month of October, 1811, a gentleman, thirty-four years of age, informed me that he had observed a white deposit in his urine during the whole of the preceding summer. He had taken considerable quantities of soda water, which he thought increased the sediment, and alkalies in any other form produced a very obvious aggravation of the complaint.

His urine was at all times clear when voided; but after a few hours, a white powder was observed to separate from it, and a film of crystalline matter formed upon the surface. The former consisted of phosphate of lime and mucus; the latter of the ammoniaco-magnesian phosphate.

He was directed to take one drachm of muriatic acid properly diluted, at divided doses, during the day; and it was proposed that he should pursue this plan for a week; but it was discontinued on the third day on account of its acting upon the bowels, and producing a frequent desire to make water.*

* In this and other instances the sulphuric and nitric acids were occasionally substituted for the muriatic; but they were found equally inadmissible.

On the 10th of October, he was advised to take two large glasses of lemonade daily, and to substitute claret for port wine, a pint of which he was in the habit of drinking daily. Under this treatment the symptoms produced by the muriatic acid subsided, but the appearance of the urine was not at first improved.

On the 20th, the film of triple phosphate formerly constantly observed in the urine began to decrease, but the white sand remained as abundant as before: he was, therefore, directed to take twenty grains of citric acid twice a-day, and to continue the use of acid drink, as formerly.

The additional acid at first disagreed with the bowels; but this effect soon ceased, and the sediment was only observed in the urine voided in the morning: he, therefore, took another dose of the acid every night. This plan was pursued with little intermission until the beginning of December. The deposition of the phosphates gradually ceased, and he remained in perfect health until the middle of May, 1812, when, after violent exercise, and taking more wine than usual, the white sand again made its appearance in great abundance; his stomach became extremely irritable; and the acids, which he had before employed with success, brought on considerable irritation in the bladder. The addition of ten drops of laudanum to each dose of the citric acid prevented this effect, and he was thus enabled to continue the acid, which in a fortnight relieved his complaint.

This gentleman informed me, that, whenever he omitted the use of an acid diet, or took much wine, especially port, his urine deposited the white sand and mucus for two or three successive days.

Case 4.—A gentleman, eighty years of age, who had twice submitted to the operation for the stone within five years, voided with his urine considerable quantities of white sand and mucus.

From the age of this patient, and the account of his case, there appeared little doubt that the calculi had been formed in consequence of a diseased prostate gland, in the manner described by Sir Everard Home;* and on examining them, they were found to contain no uric nucleus, nor indeed had there been any symptoms of disease in the kidneys, at any previous period.

This gentleman had been in the habit of taking soda water, from which he was now desired to abstain, with a view of putting him upon the acid plan of treatment. He was ordered to take eight drops of muriatic acid three times a-day in two

* Practical Observations on the Treatment of Diseases of the Prostate Gland, p. 39.

 tabletable-spoonsful of water; but the third dose produced so much irritation in the bladder, and consequent increase of his symptoms, that it became necessary to adopt another treatment.

Lemon-juice, or a solution of the pure citric acid, when given in quantity sufficient to produce any change in the appearance of the urine, had the same effect as the muriatic acid.

As water impregnated with carbonic acid could not be procured, he was directed to dissolve, in separate portions of water, twenty grains of citric acid, and thirty grains of the crystallised carbonate of potash, and to take the mixed solutions during the effervescence. This quantity was at first only taken night and morning, but as it agreed perfectly well, it was afterwards repeated four and five times daily. Under these circumstances the appearance of the urine was soon improved, and both the mucus and the sand were considerably diminished in quantity. In six weeks the urine, when voided, was transparent; but a considerable deposition of the phosphates took place, when it had remained for some hours at rest. In this state he left London, and has since informed me that the sediment gradually diminished under the use of the carbonic acid; that his urine is never turbid; and that the irritation in the bladder has entirely subsided.

It did not appear necessary to detail the minutiæ of the above cases: they have been selected with a view to elucidate the treatment of the disease, as far as it depends upon chemical principles, and to furnish the data upon which the following conclusions are founded.

1. That where alkalies fail to relieve the increased secretion of uric acid, and to prevent its forming calculi in the kidneys, or where they disagree with the stomach, magnesia is generally effectual; and that it may be persevered in for a considerable time without inconvenience, where the tendency to form excess of uric acid remains.

2. When the alkalies, or magnesia, are improperly continued, after having relieved the symptoms connected with the formation of the red sand, or uric acid, the urine acquires a tendency to deposit the white sand, consisting of the ammoniaco-magnesian phosphate and phosphate of lime.

3. The mineral acids (muriatic, sulphuric, and nitric) diminish or entirely prevent the deposition of the phosphates; but are apt to induce a return of the red gravel.

4. That vegetable acids, especially the citric and tartaric, are less liable to produce the last-mentioned effects, even when taken in large doses for a long time; and that carbonic acid is particularly useful in cases where the irritable state of the bladder prevents the exhibition of other remedies.—*Phil. Trans.*

CRITICAL

